# Corneal stromal roughness after VisuMax and Intralase femtosecond laser photodisruption: An atomic force microscopy study

**DOI:** 10.1371/journal.pone.0252449

**Published:** 2021-05-27

**Authors:** Juan Gros-Otero, Samira Ketabi, Rafael Cañones-Zafra, Montserrat Garcia-Gonzalez, Cesar Villa-Collar, Santiago Casado, Miguel A. Teus

**Affiliations:** 1 Clínica Rementería, Madrid, Spain; 2 Universidad CEU San Pablo, Madrid, Spain; 3 Hospital Universitario Príncipe de Asturias, Alcalá de Henares, Madrid, Spain; 4 Clínica Novovisión, Madrid, Spain; 5 Universidad Europea, Madrid, Spain; 6 IMDEA Nanociencia, Madrid, Spain; 7 Facultad de Ciencia e Ingeniería de Alimentos, Universidad Técnica de Ambato, Ambato, Ecuador; Singapore Eye Research Institute, SINGAPORE

## Abstract

**Purpose:**

To compare the induced corneal stromal bed roughness measured with atomic force microscopy (AFM) after LASIK flap creation with the IntraLase 60 kHz and the VisuMax femtosecond laser platforms.

**Methods:**

Three freshly enucleated porcine eyes were operated with each femtosecond laser in this experimental study. Standard LASIK treatment parameters were used for the experiment. After LASIK flap creation, the corneal stromal roughness was assessed using a JPK NanoWizard II^®^ AFM in contact mode immersed in liquid. Olympus OMCL-RC800PSA commercial silicon nitride cantilever tips were used. Surface measurements were made in 10 regions of the central cornea of each sample measuring 20 x 20 microns, at 512 x 512 point resolution. Roughness was measured using the root-mean-square (RMS) value within the given regions.

**Results:**

Measurements from 30 regions of the 3 eyes (10 measurements per eye) in the Intralase (FS1) group, and 30 regions of the 3 eyes (10 measurements per eye) in the VisuMax (FS2) group were analyzed. There was a statistically significant difference in mean ± standard deviation RMS values between the FS1 and the FS2 groups (360 ± 120 versus 230 ± 100 nm respectively; P< 0.00001).

**Conclusion:**

This AFM study indicates that the surface of the stromal bed after LASIK flap creation is smoother in the FS2 group than the FS1 group.

## Introduction

Laser in situ keratomileusis (LASIK) using a femtosecond laser has become a popular tool among refractive surgeons, as it achieves higher precision in flap thicknesses and fewer severe complications such as buttonholes or irregular ablations [1].

Globe fixation is a critical step in femtosecond laser procedures and is obtained by a suction ring attached to a certain docking structure. Traditionally, femtosecond laser platforms designed exclusively for LASIK surgery employ a flat patient interface because it is assumed that corneal flattening permits the cutting of more planar flaps. However, a significant increase of intraocular pressure (IOP) is unavoidable during flap creation using this technique [2]. With the launch of dual femtosecond laser platforms for LASIK as well as cataract surgery and/or small incision lenticule extraction (SMILE), curved interfaces were developed. Theoretically, these induce a smaller IOP rise during the procedure and create less corneal distortion [3]. To the best of our knowledge there is no published evidence supporting that this modification offers any improvement in flap morphology or refractive results. On the other hand, it has been found that flap morphology depends on the femtosecond laser platform when flat interfaces are used [4]. It has also been shown that flap irregularities induce worse postoperative visual results [5].

Atomic force microscopy (AFM) can be used to assess the roughness of a certain surface with precision that cannot be offered by currently available clinical imaging technologies, and in more physiological conditions than transmission electron microscopy [6].

IntraLase (Abbott Medical Optics Inc, Santa Ana, California) is a femtosecond laser platform that uses a flat corneal interface, induces a significant IOP rise, and emits high energy (>1μJ)—low frequency (60kHz) laser pulses. It has a non-overlapping pulse pattern, and induces microplasma bubbles, with a diameter ranging from 1 to 5 microns that might overlap with adjacent laser spots. In addition, it is by far the most studied femtosecond laser platform.

On the other hand, VisuMax (Carl Zeiss Meditec AG, Jena, Germany) is a femtosecond system with a curved patient interface, which induces a mild IOP elevation, and uses a low energy (<300 nJ)—high frequency (200kHz) laser configuration for flap creation. It’s a “real non-overlapping” pulse pattern because the cavitation bubbles are <1 micron, so the gas seems to remain within the same area of the laser spot diameter. This laser system can be used both for LASIK flap creation and for SMILE procedure.

The aim of the current study was to evaluate stromal roughness after corneal flap creation with the two aforementioned lasers using AFM. We hypothesized that significant differences in surface roughness could be observed as a result of differences in numerous technical characteristics between these two platforms.

## Materials and methods

We designed an experimental study that included 6 freshly enucleated porcine eyes. All eyes were retrieved within 3 hours after the animals were sacrificed in a licensed slaughterhouse (Matadero Comarcal del Barbanza, Ribeira, Spain), and were stored at 4°C before the experiments. Exclusion criteria were the presence of conjunctival lesions that could interfere with femtosecond laser docking, corneal opacities and intraoperative complications. No statements for the use of animals in ophthalmic research are applicable, as the eyes were purchased in a licensed abbatoir (Matadero Comarcal del Barbanza, Ribeira, Spain), and were obtained from animals slaughtered for human consumption in the cited licensed abattoir.

### Surgical procedure

The IntraLase^®^ 60 kHz femtosecond laser (Abbott Medical Optics, Inc.) was used for the creation of a LASIK flap in three eyes (FS1 group), and the VisuMax^®^ femtosecond laser (Carl Zeiss v.2.10.7) was used in another 3 eyes (FS2 group). Standard parameters were used in all cases (Table 1).

**Table 1:** LASIK photodisruption parameters for Intralase (FS1) and VisuMax (FS2).

	FS1	FS2
Flap thickness	110 microns	130 microns
Flap diameter	9 mm	9 mm
Hinge position	90°	90°
Hinge angle	50°	50°
Sidecut angle	135°	90°
Bed energy	0.95 mJ	1 90 nJ
Spot separation	8 microns	6 microns
Track separation	6 microns	4.5 microns
Ablation time	18 seconds	19 seconds

In all cases, after the flap was cut, it was first lifted and then put back into its position using a LASIK spatula. Then, a cap that contained the cornea and a 2 millimeters wide scleral rim was dissected free from each eye using Westcott scissors and all choroidal tissue was meticulously stripped from the corneo-scleral rim. Next, the corneas were trephined without lifting the flap using a 9 mm wide Barron vacuum donor cornea button punch. After the corneo-scleral rim was discarded, the flap was carefully lifted from the corneal button using corneal forceps without touching the underlying stroma under a surgical microscope. Then, the corneal button was glued with cyanoacrylate adhesive on its endothelial side on a microscope slide, transferred to a petri dish and covered with 2.5% glutaraldehyde solution until AFM measurements were performed.

All surgical procedures were performed in the same day by the same experienced refractive surgeon (JGO).

### Imaging

We routinely perform our atomic force imaging using a JPK NanoWizard II^®^ AFM coupled to a Nikon Eclipse Ti-U inverted optical microscope, in contact mode immersed in liquid, using Olympus OMCL-RC800PSA commercial silicon nitride cantilever tips (0.05 N/m, 18 kHz), with typical 15 nm radius at the end. Vertical accuracy of the instrument is in the order of 0.1 nm.

In each sample, surface measurements were made in 10 areas of the central corneal stroma with dimensions 20 μm x 20 μm, at 512 x 512 point resolution. Images were processed and analyzed using the JPK Data Processing software (JPK Instruments AG, Berlin, Germany). We measured surface roughness using the root-mean-square (RMS) value within the given areas. Measuring force was calibrated at the setpoint before measurements and the calibrated normal forces established for each measurement included in this paper can be found in S1 Table. In addition, surface preservation was controlled not only by the force values, but also by the trace and retrace image comparison, by doing so we obtained evidence that sample nanostructure is mainly conserved during the probe and that the RMS values yielded are (and as a result) the same. All measurements were made by the same experienced AFM scientist (SC). The Wilcoxon signed-rank test was used to compare the roughness obtained for each femtosecond laser group.

## Results

**Fig 1:**
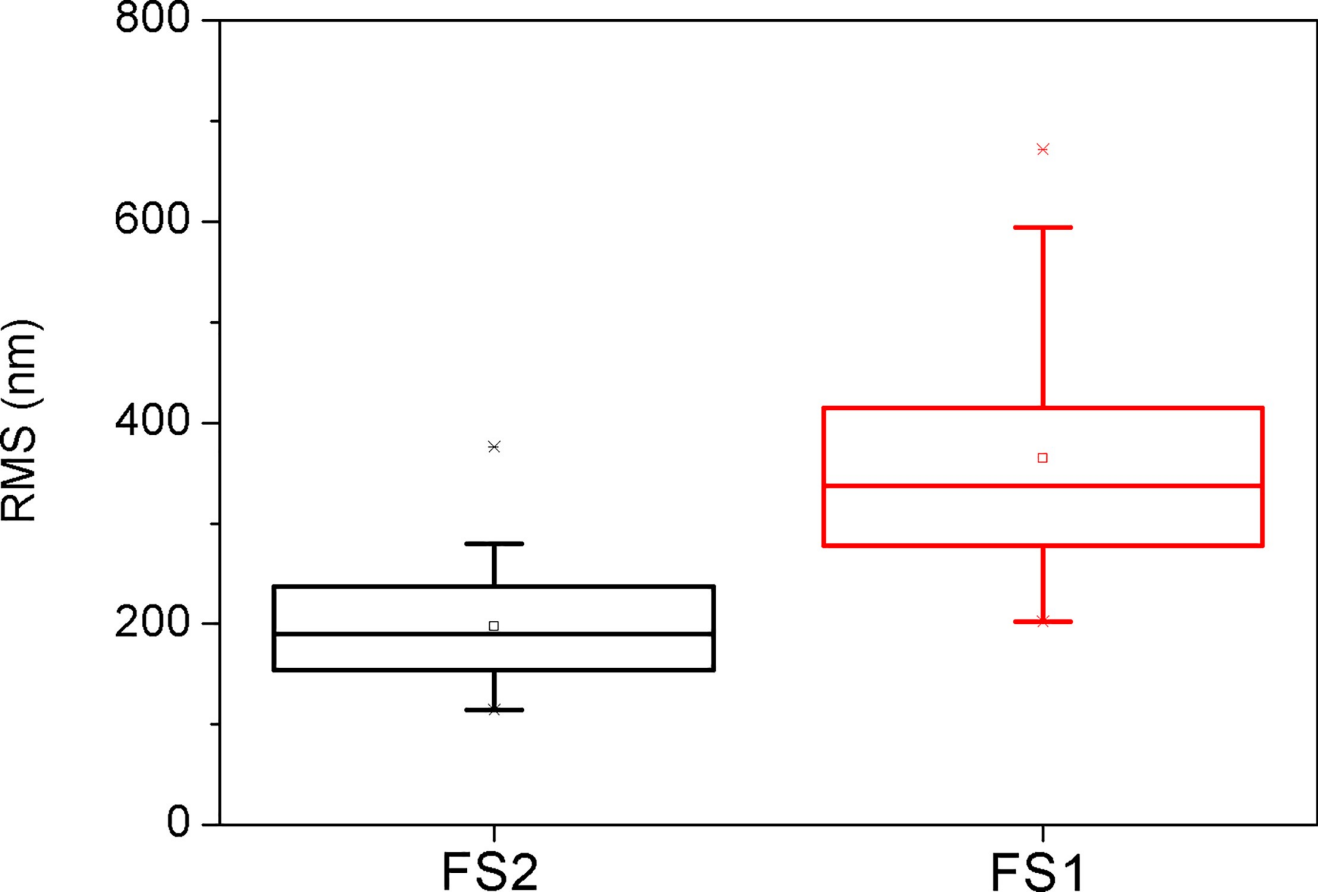
RMS comparison between FS1 and FS2.

**Fig 2:**
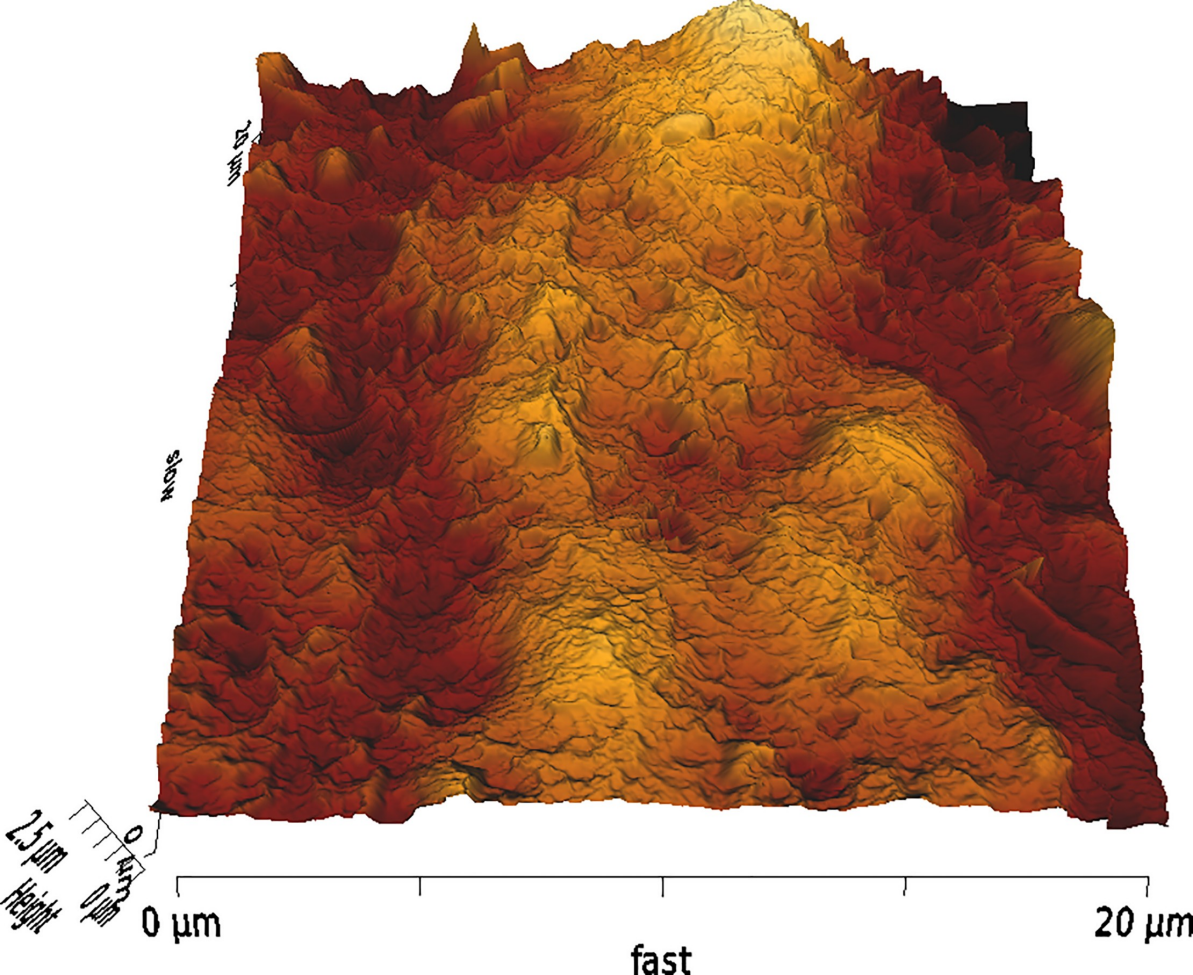
Example image of corneal surface treated with FS1 with 3D AFM topography.

**Fig 3:**
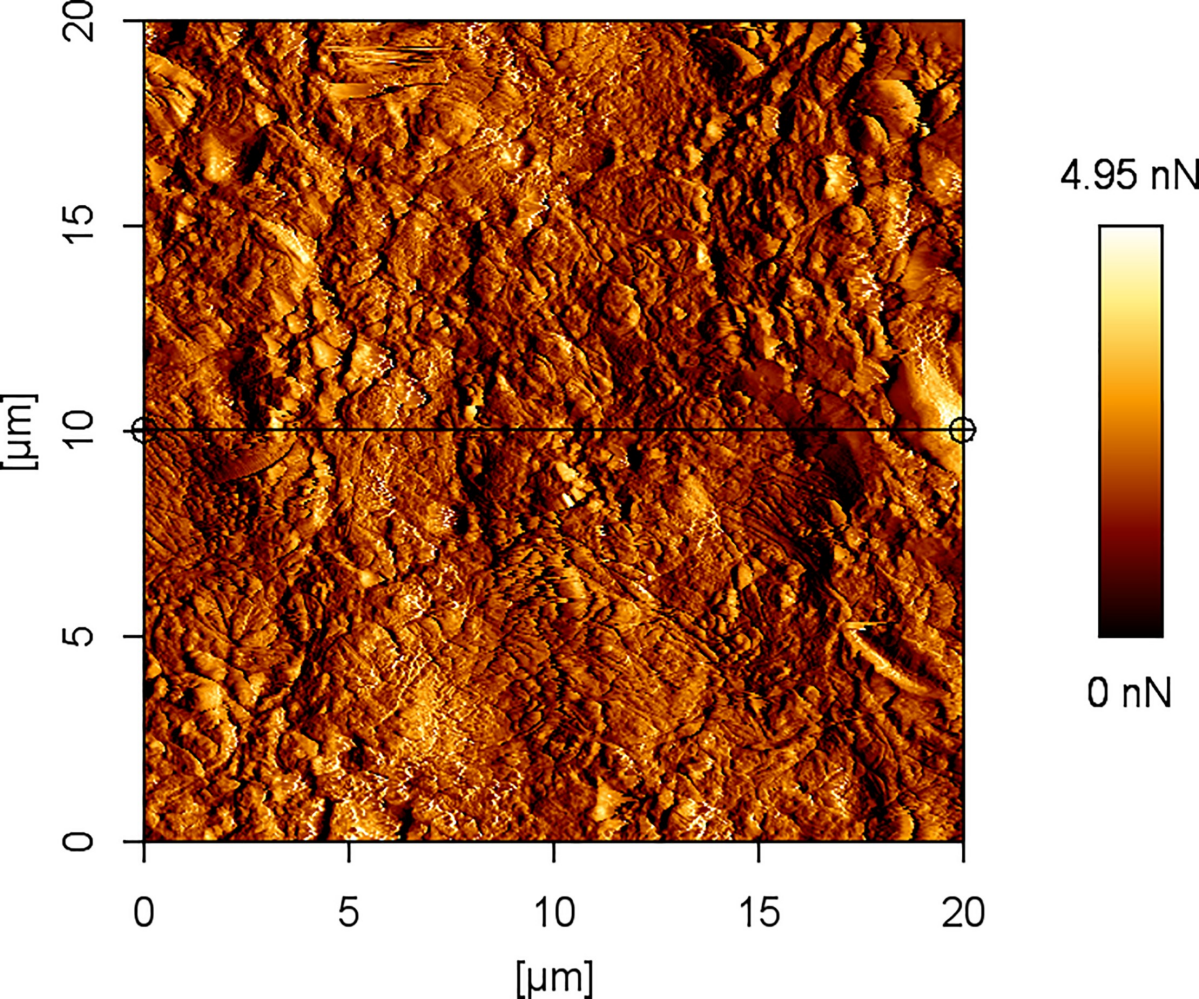
Example image of corneal surface treated with FS1 with AFM vertical deflection.

**Fig 4:**
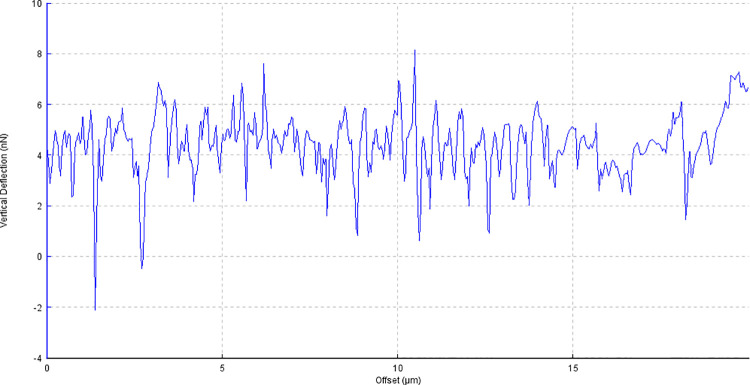
Example image of corneal surface treated with FS1 with AFM line profile.

**Fig 5:**
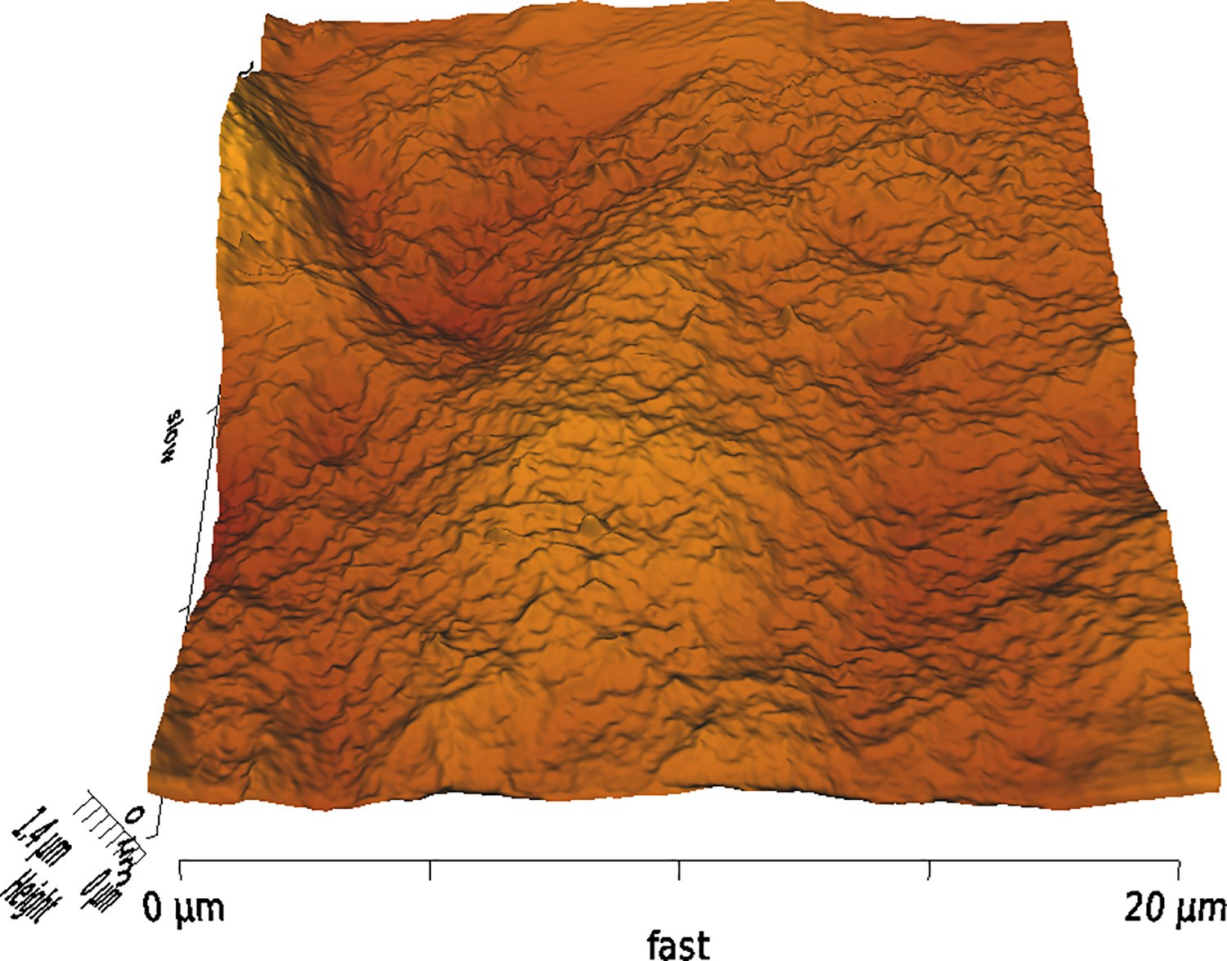
Example image of corneal surface treated with FS2 with 3D AFM topography.

**Fig 6:**
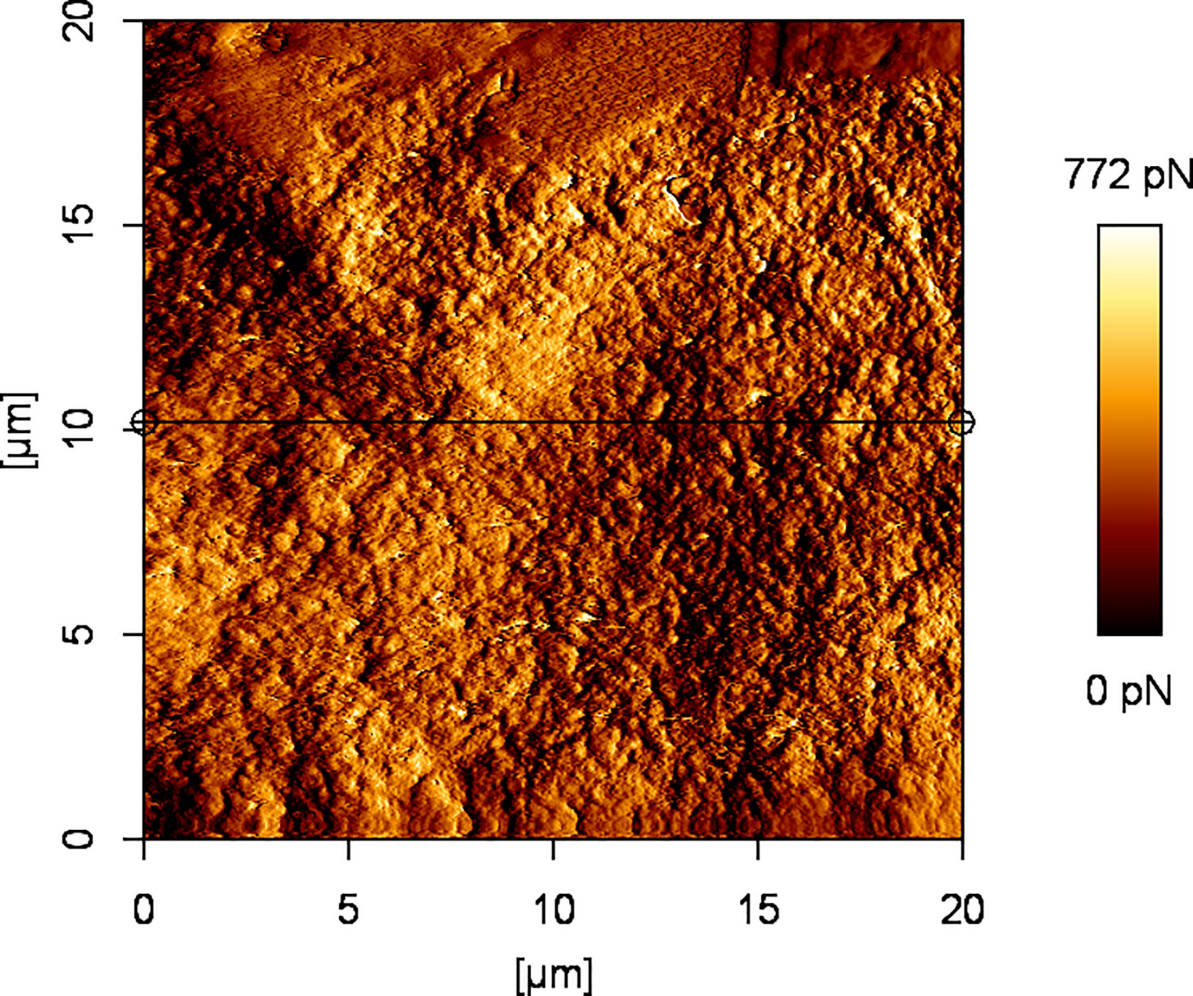
Example image of corneal surface treated with FS2 with AFM vertical deflection.

**Fig 7:**
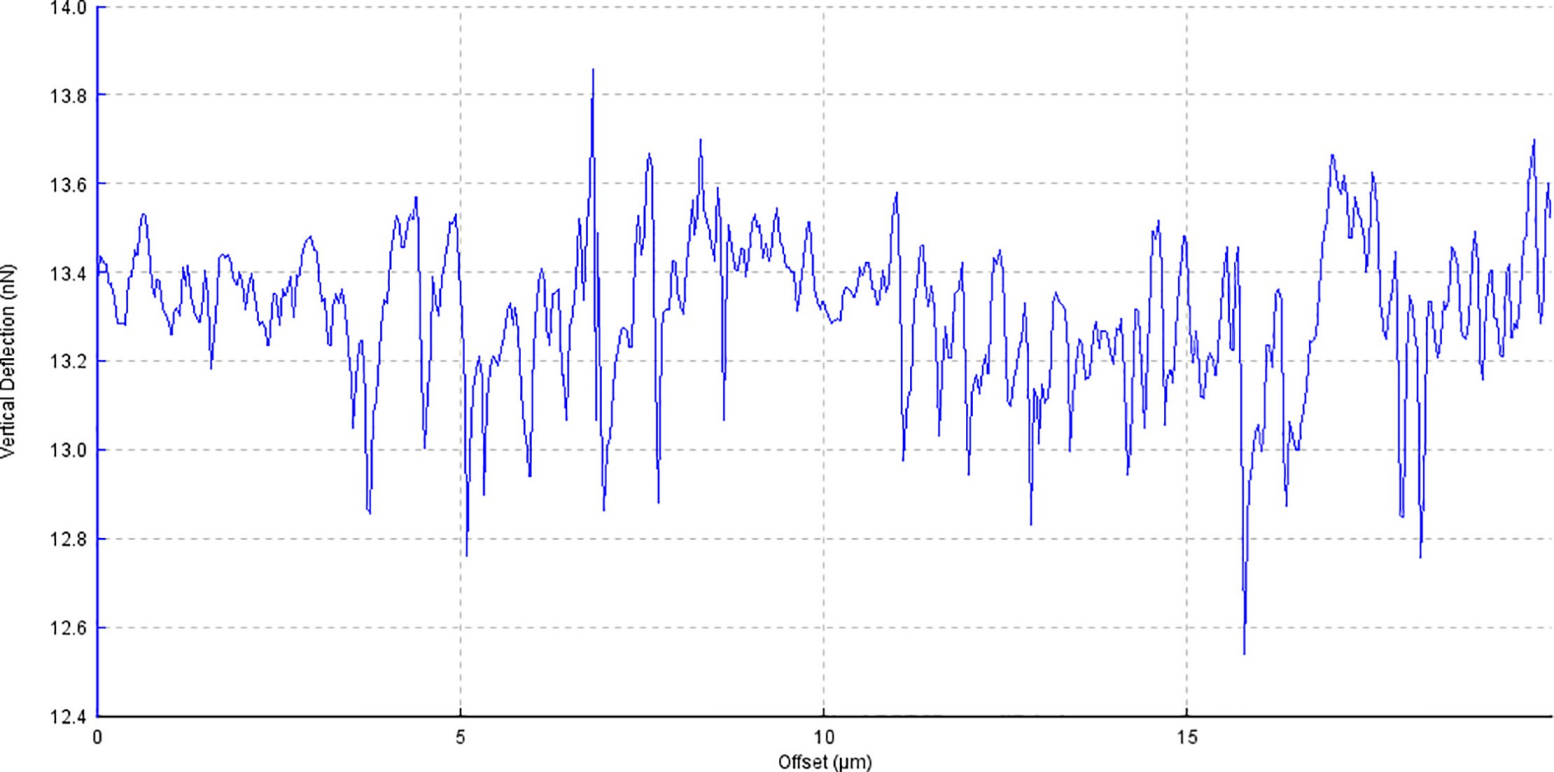
Example image of corneal surface treated with FS1 with AFM line profile.

We studied 30 regions from 3 eyes (i.e. 10 measurements per eye) in the FS1 group, and 30 regions from 3 eyes (i.e. 10 measurements per eye) in the FS2 group. All AFM measurements were taken within less than 3 days after the trephination. RMS values were calculated from the three-dimensional AFM images taken from each sample. Mean ± standard deviation of RMS values are 360 ± 120 nm for the FS1 group, and 230 ± 100 nm for the FS2 group (P< 0.00001, Fig 1). RMS values in nanometers of each 20x20 area measured are included in Table 2. Figs 2–7 are image examples of the areas studied.

**Table 2:** Root mean square values (nanometers) of each 20x20 area measured.

IntraLase sample 1	481	208	292	202	230	288	250	278	327	506
IntraLase sample 2	312	312	346	337	338	277	312	376	275	258
IntraLase sample 3	396	414	419	672	358	415	408	504	558	510
VisuMax sample 1	158	206	143	220	280	257	188	154	191	259
VisuMax sample 2	126	376	172	268	191	123	188	114	131	237
VisuMax sample 3	243	161	151	217	139	279	175	199	185	204

## Discussion

In our study, AFM evaluation indicated that the stromal beds of eyes in the FS2 group were smoother compared to the ones in the FS1 group. These differences are also obvious in the three dimensional images generated by AFM.

The issue of corneal bed roughness following LASIK flap creation has been investigated using high-magnification microscopy. Kymionis et al [8], used scanning electron microscopy (SEM) to compare stromal bed roughness obtained with the use of two different femtosecond laser platforms and a mechanical microkeratome. These authors found that in comparison to the mechanical microkeratome, femtosecond lasers produced smoother stromal beds [8]. No statistically significant differences in stromal bed smoothness were found when the femtosecond devices were compared with each other [8]. In contrast, our work focuses on the analysis of the stromal roughness between two femtosecond lasers that have substantial differences in critical parameters such as energy and docking system.

Sarayba et al [9], also used SEM to compare stromal roughness following corneal flap creation with the IntraLase 15 kHz, the IntraLase 30 kHz and the Hansatome mechanical microkeratome. These investigators reported lower stromal roughness when the IntraLase 30 kHz device was used compared to the Intralase 15 kHz device and the mechanical microkeratome.

In the studies by Kymionis et al [7] and Sarayaba et al [8], SEM was used for the assessment of corneal bed roughness. On the contrary, our study employed AFM, thus allowing for an analysis at a sub-nanometer scale. In addition, the results obtained with AFM are based on a three-dimensional analysis and allows quantitative analysis, while results obtained with SEM are based on two-dimensional analysis and do not allow this quantitative comparison [9].

Our group recently reported [10] lower RMS values after LASIK flap creation with LenSx compared with IntraLase iFS150 kHz. Compared to that data, VisuMax obtained the smoothest corneal stromal beds (lower RMS values).

The two femtosecond laser platforms analyzed in the current study differed remarkably in several aspects. These dissimilarities in technical characteristics could explain the differences observed in our investigation. Firstly, the laser parameters that we used for LASIK flap creation are considered standard settings for each platform (Table 1). However, these settings are not identical for the two devices. A second important technical difference between the two platforms is gas management: the IntraLase system creates a pocket that serves as a trap for the gas liberated during the relatively slow corneal tissue photodisruption. On the other hand, the VisuMax platform does not use such a system because the photodisruption is faster and therefore gas accumulates too slowly to interfere with tissue cutting. A third important difference between the two devices is the geometry of the corneal interface: a curved interface presumably causes less tissue distortion since it is designed to match the natural curvature of the cornea. On the other hand, conventional flat cones induce significant tissue distortion due to flattening of the cornea during photodisruption. These transient anatomical changes might affect the interaction between the laser beam and the corneal tissue, and might also interfere with the gas or plasma evacuation during the procedure.

All these considerations are relevant only for the first step of the femtosecond LASIK procedure, i.e. the creation of the flap. In clinical practice, after the LASIK flap is lifted, the excimer laser is applied on the corneal stroma to achieve refractive correction. The ablation by the excimer laser can theoretically smoothen the roughness created by femtosecond laser photodisruption.

Interestingly, a smoother stromal bed was produced during LASIK flap creation by the FS2 system, (i.e. by a device used for SMILE surgery) but not by the FS1 system, which is the most widely studied femtosecond laser for LASIK surgery [4, 5]. Interface stromal roughness has been described as a potential risk factor for delayed visual acuity recovery after SMILE surgery [11], but interestingly, our data show that eyes in the FS2 group had smoother corneal stroma surfaces than eyes in the FS1 group. Thus, it might well be that the absence of the smoothing effect of the excimer laser ablation in SMILE allows the original roughness to remain as a potential problem (in contrast with LASIK), and thus making the femtolaser induced stromal roughness more troublesome after SMILE.

In conclusion, in this experimental study, atomic force microscopy shows that the VisuMax femtosecond laser produces smoother stromal bed following LASIK flap creation than the IntraLase femtosecond laser. Probably, the effect of the stromal roughness will be minimal on both the refractive and vision quality results, and in the case of LASIK, the smoothing effect of the excimer ablation of one of the stromal surfaces, will probably decrease this potential effect even further.

## Supporting information

S1 Table Calibrated normal forces established at the setpoint on each measurement in nanoNewtons. (DOCX)
