# Supplementary material for: Corneal stromal roughness after VisuMax and Intralase femtosecond laser photodisruption: An atomic force microscopy study
Source: PLoS One. 2021 May 27;16(5):e0252449. doi: 10.1371/journal.pone.0252449 (PMC8158881; doi:10.1371/journal.pone.0252449)
Supplement: S1 Table — (DOCX) [file pone.0252449.s001.docx]

**S1 Table. Calibrated normal forces established at the setpoint on each measurement in nanoNewtons.**

| IntraLase sample 1 | 07 | 07 | 12 | 15 | 07 | 07 | 07 | 07 | 07 | 07 |
| --- | --- | --- | --- | --- | --- | --- | --- | --- | --- | --- |
| IntraLase sample 2 | 07 | 07 | 07 | 07 | 07 | 07 | 07 | 07 | 07 | 07 |
| IntraLase sample 3 | 07 | 06 | 06 | 06 | 06 | 06 | 07 | 07 | 08 | 08 |
| VisuMax sample 1 | 07 | 07 | 07 | 07 | 07 | 07 | 07 | 07 | 07 | 07 |
| VisuMax sample 2 | 07 | 07 | 07 | 07 | 07 | 07 | 12 | 12 | 12 | 12 |
| VisuMax sample 3 | 07 | 07 | 07 | 07 | 07 | 07 | 07 | 07 | 07 | 07 |
